# Imbalance of the Gut Microbiota May Be Associated with Missed Abortions: A Perspective Study from a General Hospital of Hunan Province

**DOI:** 10.1155/2021/5571894

**Published:** 2021-12-20

**Authors:** Bingsi Gao, Xingping Zhao, Xinyi Liu, Xuan Yang, Aiqian Zhang, Huan Huang, Yu-ligh Liou, Dabao Xu

**Affiliations:** ^1^Department of Obstetrics and Gynecology, The Third Xiangya Hospital of Central South University, 138 Tongzipo Rd, Changsha, 410013 Hunan, China; ^2^Department of Obstetrics and Gynecology, Hunan Provincial Maternal and Child Health Hospital, 53 Xiangchun Rd, Changsha, 410008 Hunan, China; ^3^Central South University, Xiangya School of Medicine, 172 Tongzipo Rd, Changsha, 410013 Hunan, China; ^4^Xiangya Medical Laboratory, Central South University, Changsha, Hunan 410078, China; ^5^The First Affiliated Hospital of Guangdong Pharmaceutical University, Guangzhou, China

## Abstract

**Objective:**

To conduct a preliminary investigation that shows the possible correlation between the change of gut microbiota and missed abortions (MAs), which further provides a new potential insight for the prevention and therapy of MAs.

**Method:**

One hundred women, including 50 patients with MAs (case group) and 50 normal pregnant women (control group), were enrolled in the study. Fecal specimens were collected in the first trimester. Bacterial DNA was extracted, hybridized with primers of specific genes, and then detected by bacterial chip. The composition and the relative abundance of the gut microbiota were compared and analyzed. Furthermore, Kyoto Encyclopedia of Genes and Genomes enrichment analysis was used to explore the relative pathways.

**Results:**

(1) The *α*-diversity and *β*-diversity of the gut microbiota in patients with MAs were significantly lower than that those in normal pregnant women (*P* < 0.05). At the phylum level, *Firmicutes*, *Proteobacteria*, *Actinomycetes*, and *Bacteroidetes* accounted for the main proportion of intestinal flora in the 2 groups. Only *Actinobacteria* was high in the case group. Significant differences were found between the two groups at the phylum level (*P* < 0.05). *Prevotella*, *Lactobacillus*, and *Paracoccus* were significantly more abundant in the control group than in the case group at the genus level (*P* < 0.05). (2) KEGG pathway enrichment analysis found significant differences in 27 signaling pathways and metabolic pathways between the two groups of differentially expressed genes (all adjusted *P* < 0.05). (3) The positive rate of *M. hominins* (MH) detection in the control group was significantly higher in the MA group (*χ*^2^ = 7.853, *P* = 0.004).

**Conclusion:**

The high abundance of *Actinobacteria* in the MA group was the first time found and reported in the study. The dysbiosis of the gut microbiota correlates with MAs. This study provided insights into the potential change of gut microbiota of MAs and the potential underlying mechanisms through certain impaired lipid metabolism and aroused inflammation pathways. Comprehensive insights regarding gut microbiota may facilitate improved understanding and the development of novel therapeutic and preventive strategies for MAs.

## 1. Introduction

Miscarriage, common in pregnant women, can cause physical and psychological harm, such as excessive bleeding, infection, anxiety, depression, and posttraumatic stress disorder. Missed abortions (MAs) are a kind of miscarriage, which refers to the stagnation of embryonic or fetal development with no cardiac activity, still staying in the uterine cavity for several days or weeks, and cannot be discharged naturally in time. It is defined as a pregnancy that fails to develop as a result of the cervix remaining closed [1]. MAs account for over 15% of spontaneous miscarriages and show an increasing trend [2]. Maternal factors, genetic and uterine abnormalities, endocrine and immunological dysfunctions, infections, and nutritional and environmental factors have been reported as risk factors for MAs [3–5]. However, approximately 30% causes of MAs remain unclear [6].

In recent years, an increasing number of studies have emerged indicating that the gut microbiota is involved in host endocrine metabolism, innate and acquired immunity maturation, epithelial cell injury modulation, and energy balance through various hormones or regulators [7–13]. The host provides nutrition and habitat for microbial communities and benefits from its symbionts that contribute to trophic functions, defensiveness, and metabolism [14]. In turn, the gut microbiota also plays a role in modulating the host response through metabolic activities [15, 16]. More and more evidence has revealed that the human gut microbiota is closely related to human health and diseases [17, 18], including obesity, type II diabetes, polycystic ovarian syndrome, and the processing of chronic infections [19–23]. This concept of gut microbiota disease provides a new exploration direction for the possible unknown risk and pathogenesis of MAs. To date, numerous investigations have reported that changes of the vaginal microbiota, such as bacterial imbalance, *Mycoplasma* infection, *Candida* infection, and viral infection, are associated with miscarriage in the first trimester [5, 24–27]. However, few studies have explored the potential relationship or deciphered the interplay between the gut microbiota and MAs.

This study hypothesized that the change of gut microbial communities might regulate the local gut microenvironment and the host metabolism, resulting in the possible outcome of MAs.

## 2. Materials and Methods

### 2.1. Study Participants

This study was approved by the ethics committee of the Third Xiangya Hospital of Central South University (IRB No. 2019-S460). From January to December 2019, fifty patients diagnosed as MAs in the first trimester (pregnancy within 12 weeks) without forwarding surgery were enrolled for the case group. For the control group, 50 pregnant women of similar age and a similar volume of menstrual flow to those of the case group before pregnancy were enrolled. They still had normal regular obstetric checkup results in the first trimester and had the normal pregnancy or birth outcome. All participants were enrolled and all informed consent was signed. According to the guidance or regulations of the Third Xiangya Hospital, the regular times and items of obstetric examination and treatment of pregnant women were implemented for all women in the 2 groups.

All participants satisfied the following inclusion criteria: (1) women aged 18–45 years who are healthy in the first-trimester pregnancy and had a history of normal menstrual cycles; (2) mentally competent participants able to understand the consent form and communicate with study staff; (3) no history of chronic diseases such as diabetes, enteritis, hepatitis, or tuberculosis; (4) no use of antibiotics in the lead up to the collection of specimens; and (5) no history of smoking, drinking, or toxic exposure. The exclusion criteria included (1) patients with cervical or intrauterine cancers or diseases, such as cervical cancer, genital tract malformations, submucosal fibroids, adenomyosis, and uterine adhesions; (2) patients with heart, liver, or kidney failure; (3) patients with inability to understand and cooperate with hospital staff due to mental impairment; (4) patients who withdrew from the study; and (5) patients with no signed informed consent or routine examinations uncompleted.

**Figure 1:**
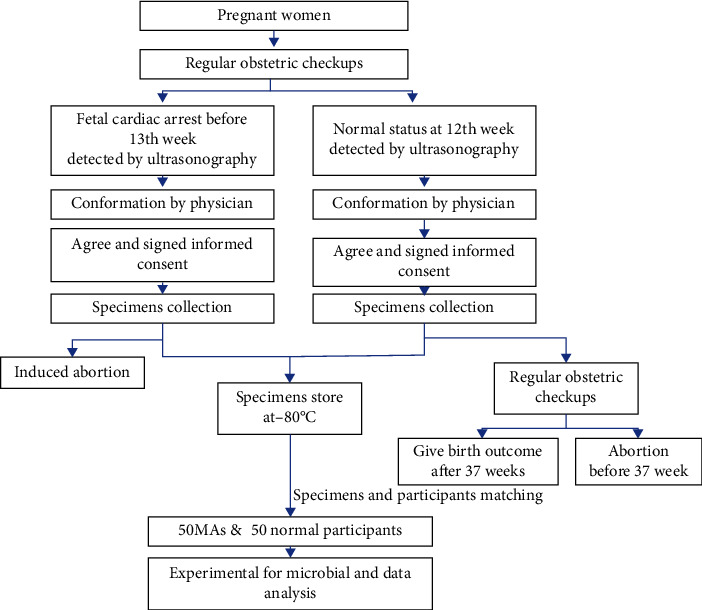
Procedures used in the study. Mas: missed abortions.

Fecal specimens from all participants were collected in the first trimester of pregnancy (before or in the 12^th^ week) and stored at -80°C for further investigations. The procedure of the study is showed in Figure 1.

### 2.2. Bacterial DNA Extraction and Labeling

Bacterial DNA was extracted from the fecal specimens using the Stool DNA Extraction Kit (Halgen, Ltd., Guangzhou, China) following the manufacturer's instruction. The primers F44 (RGTTYGATYMTGGCTCAG) and R1543 (GGNTACCTTKTTACGACTT) were used to amplify the V1–V9 regions of the 16S rRNA. Approximately 20–30 ng of the extracted DNA was used in a 50 *μ*l polymerase chain reaction (PCR) according to the following conditions: an initial denaturing step at 94°C for 3 minutes, followed by 30 cycles of 94°C for 30 seconds, 55°C for 30 seconds, 72°C for 60 seconds, and a final extension step of 72°C for 3 minutes. Agarose gel electrophoresis was performed to confirm the success of the PCR amplification. The DNA products amplified by PCR were directly labeled with a DNA labeling kit (Hagen Co., Ltd., Guangzhou, China) without purification. 5 *μ*l PCR qualified DNA were loaded to the slide of Hagen array and mixed with hybridization buffer for hybridization according to the manufacturer's instruction.

### 2.3. Microarray Hybridization

Probes were selected from all the variable regions of the bacterial 16S rRNA. Each probe was designed to be approximately 40 bp in length. The hybridization mixture is contained 500 ng Cy5-labeled test sample DNA and 50 ng Cy3-labeled pool reference DNA for microorganism array (Halgen Ltd., Guangzhou, China). Add hybridization buffer to a final volume of 150 *μ*l in the hybridization mixture, then heat to 100°C for 5 minutes, and cool on ice for 5 minutes. The mixtures were then placed in a slide of hybridization box and hybridized in an oven for 3.5 hours at 37°C. Slides were washed in 1x Saline Sodium Citrate (SSC), 0.25% Triton X-100, 0.25% sodium dodecyl sulfate (SDS), and 1x Dye Protector for 15 minutes at 63°C and then rinsed in 1x Dye Protector until water droplets cleared from the slides immediately following withdrawal from the solution. Slides were scanned promptly using a dual-channel scanner.

### 2.4. Data Analysis for Microorganisms

The relative abundance of each intestinal microbiota is proportional to the average value of its specific probe Cy5/Cy3 ratio. Therefore, the type of intestinal microbiota could be determined by the ratio of each probe Cy5/Cy3. The sequencing program (Halgen, Ltd., Guangzhou, China) was used to detect the species and relative abundance of microorganisms in the fecal specimen. The alpha (*α*) and beta (*β*) diversity calculations and the QIIME (Quantitative Insights into Microbial Ecology) software default original parameters were used to analyze the general characteristics of the intestinal microbiota. The Wilcoxon rank-sum test was used to calculate the *α*-diversity within each group and the differences in *β*-diversity among groups. Principal coordinate analyses (PCoA) and nonmetric multidimensional scaling (NMDS) analyses were performed using the QIIME module and visualized in R (version 3.5.2). The *α*-diversity refers to the diversity of the microbial communities in the samples of the case and control groups. The species information index, Chao, abundance-based coverage estimator (ACE), Shannon, and Simpson indexes, were used for analysis. The Chao and ACE indexes, indicating the number of microbial communities in a single sample without involving the relative abundance of each bacterial community, represented the abundance of the communities in each sample. The Shannon and Simpson indexes were coaffected by sample richness and uniformity. The sample diversity was positively correlated with the Shannon index and negatively related to the Simpson index.

First, linear discriminant analysis (LDA) and the LEfSe (linear discriminant analysis effect size) software were used to screen the significant differential microorganisms between the case and control groups. Subsequently, enrichment analysis was performed to determine the Kyoto Encyclopedia of Genes and Genomes (KEGG, version 37) metabolic pathways related to the differentially expressed genes (*P* < 0.05), together with the metabolic pathways that were significantly altered under experimental conditions, including biochemical pathways and signal transduction pathways.

### 2.5. Statistical Analysis

Statistical analyses were performed using the SPSS 22.0 software. The nonparametric tests were performed using the Kruskal–Wallis test and the Wilcoxon test. The relative abundance between groups was compared, and differential species selection was performed with the LEfSe software using LDA. A corrected *P* value of <0.05 was considered as being statistically significant. To reduce the influence of overfitting, the leave-one-out test was used for cross-validation in the random clustering algorithm.

## 3. Results

### 3.1. Baseline Characteristics of the Study Population

The basic characteristics of the 100 participants (case and control groups) were analyzed and compared in this study. The average age of the case group and the control group was 31.7 ± 4.6 years and 30.9 ± 4.1 years, respectively. A similar volume of menstrual flow history was evaluated in 46 out of 50 (92%) women in the case group and 48 out of 50 (96%) women in the control group. Pregnant women with lower menstrual volume history were observed in 4 cases (8%) of the case group and 2 cases (4%) of the control group. In the case group, 25 cases (50%) had a successful natural birth history, and 14 cases (28%) were cesarean section with live birth history. In the pregnancy history of the control group, 28 cases (56%) had a successful natural birth history and 15 cases (30%) were cesarean section with live birth history. There were no significant differences in any of the parameters of characteristics between the 2 groups (*P* > 0.05) except for the number of previous abortions and history of uterine cavity surgery (Table 1). These 2 factors are closely related to intrauterine adhesion (IUA), which commonly presents a reduction of menstrual flow. However, since there was no significant difference in the volume of menstrual flow between the two groups; all data were considered matched and comparable.

### 3.2. The Diversity of Gut Microbiota

**Figure 2:**
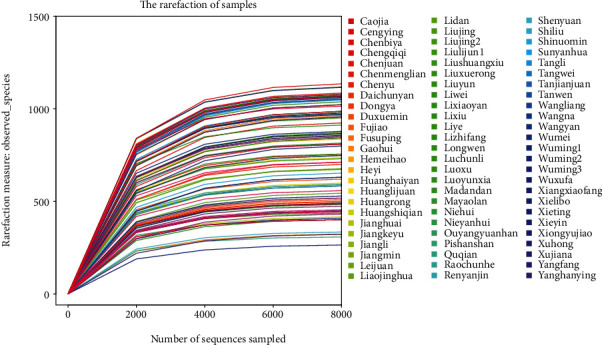
Dilution curve of each sample. The abscissa represents the number of sequences, and the ordinate represents the number of OTUs observed. According to the endpoint of the extension of the sample curve, the number of sequencing samples was found to correspond to the abscissa.

Both the *β*-diversity and the *α*-diversity of the gut microbiota were investigated following successful validation of the sequencing quality (Figure 2). The results showed that the detection serial dilution curves of all subjects (in both case and control groups) gradually changed from a significant increase to a certain level and finally reached saturation.

#### 3.2.1. *β*-Diversity of the Gut Microbiota

This result was calculated and recorded after 100 repeats of random sampling. The *β*-diversity demonstrated the significant differences between groups and can be compared using the algorithm of nonmetric multidimensional scaling (NMDS) and/or principal coordinate analysis (PCoA).

**Figure 3:**
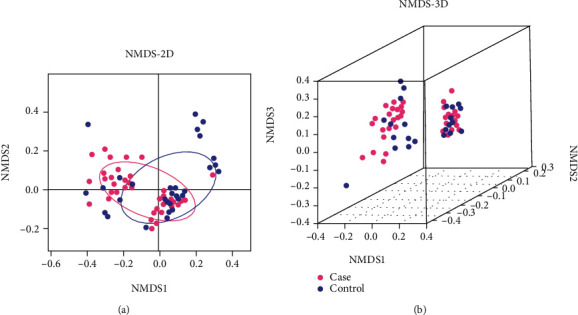
The analysis of NMDS by 2D and 3D models. (a) Two-dimensional map. (b) Three-dimensional map. The different *β*-diversity distance matrices were compared and analyzed from the original data. The 2-dimensional and 3-dimensional maps of the sample space location were obtained. It indicates an obvious difference between the case and control groups when the same color samples are grouped by circle. Each dot represented one sample by pink (case group) or blue (control group) in the map. NMDS: nonmetric multidimensional scaling.

NMDS analyses showed the case and control groups could be clearly distinguished (pink and blue are grouped separately) in Figure 3. There was a significant deviation in the confidence ellipse between the 2 groups (*P* = 0.003), implying that the diversity of the case group and the control group was significantly separated (*P* < 0.05).

**Figure 4:**
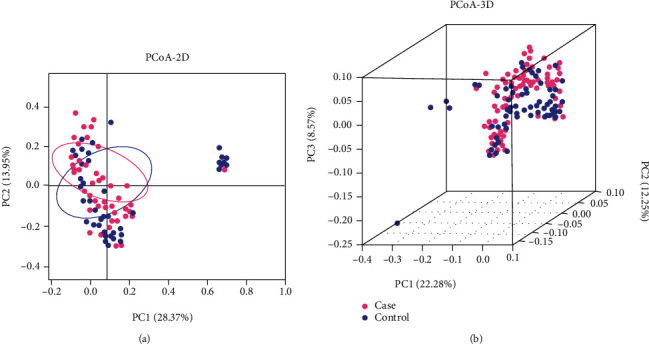
The analysis of PCoA by 2D and 3D models. (a) Two-dimensional map. (b) Three-dimensional map. PCoA map can be defined based on the distance matrix between samples to observe the differences of microbial populations between samples. If the abundance of the gut microbiota of the 2 samples is similar, the distance between the 2 points on the PCoA map will be closer. If the sample has good repeatability, the circle area is small; otherwise, the color area is large. Each dot represented one sample by pink (case group) or blue (control group) in the map. PCoA: principal coordinate analysis.

Combined with the QIIME software, the PCoA 2-dimensional and 3-dimensional maps of the *β* matrix were obtained (Figure 4). The results showed that the *β*-diversity of each sample in the 2 groups was similar, but the distance between the samples was relatively long. The confidence ellipse of the two groups has a significant deviation (*P* = 0.001), suggesting that the diversity of the case group and the control group was significantly separated (*P* < 0.05). The color area of the 2 sets of samples is small, indicating satisfactory repeatability for the sampling.

NMDS and PCoA results of the beta diversity analysis revealed that the gut microbiota in subjects with missed abortion clustered significantly compared to the controls.

#### 3.2.2. The *α*-Diversity of the Gut Microbiota

**Figure 5:**
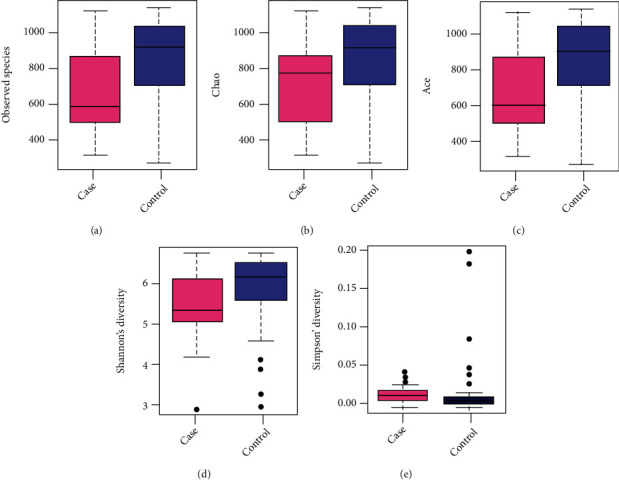
The *α*-diversity box plots of the 2 groups: (a) the Sobs; (b) Chao; (c) ACE; (d) Shannon; (e) Simpson is a type of *α*-diversity index. Each dot represented one sample by pink (case group) or blue (control group) in the map. Sobs: number of observed species; ACE: abundance-based coverage estimator.

From Sobs, Chao, ACE, and Shannon index, the comparison of *α*-diversity revealed that the diversity in the fecal specimen of the control group was significantly higher than that in the case group (*P* < 0.05), indicating a lower richness and evenness of gut microbiota in patients with missed abortion than in normal pregnant women (Figure 5 and Table 2).

### 3.3. Composition of the Gut Microbiota

In this study, the gut microbiota was analyzed at 6 levels, including the phyla, class, order, family, genus, and species.

**Figure 6:**
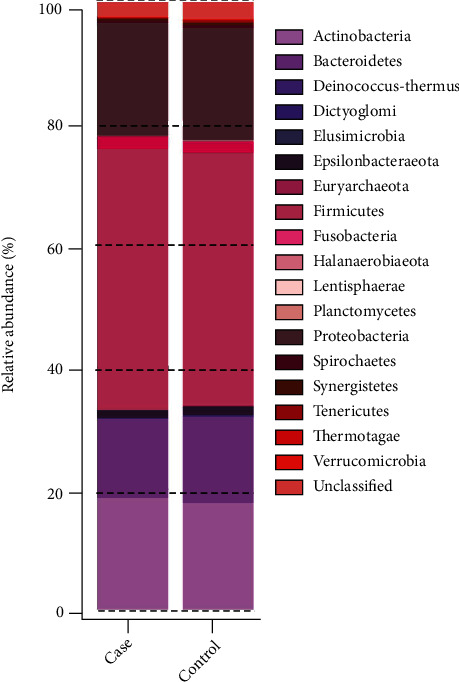
A column chart shows the relative abundances of species at the phylum level.

**Figure 7:**
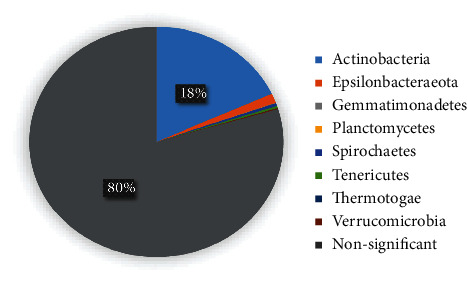
The proportion of 8 significant different phyla categories between the 2 groups. *Actinobacteria* (18.15%), *Epsilonbacteraeota* (1.32%), *Spirochaetes* (0.43%), *Tenericutes* (0.30%), *Gemmatimonadetes* (0.02%), *Planctomycetes* (0.04%), *Thermotogae* (0.01%), and *Verrucomicrobia* (0.14%).

The highest abundance of gut microbiota for phylum categories in the 2 groups was *Firmicutes*, followed by *Proteobacteria*, *Actinobacteria*, and *Bacteroidetes*; all of them were not significantly different between the two groups except *Actinobacteria* (Figure 6). Meanwhile, a total of 8 phyla, only *Actinobacteria* was high in the case group, were found to have significant differences between the two groups at the phylum level (*P* < 0.05, Figure 7). The relative abundance of *Actinobacteria* and *Epsilonbacteraeota* was >1%, and the relative abundance of the other 6 phyla (*Planctomycetes*, *Tenericutes*, *Thermotogae*, *Spirochaetes*, *Verrucomicrobia*, and *Gemmatimonadetes*) was <1%. The abundance of *Fusobacteria*, *Elusimicrobia*, *Actinobacteria*, *Firmicutes*, and *Proteobacteria* was higher in the MA group (*P* > 0.05). *Firmicutes/Bacteroidetes* ratio has been suggested as an indicator of several pathological or obese conditions [28]. The ratio was 3.29 in the case group and 2.92 in the control group.

There were 168 genera of bacteria with significant differences (*P* < 0.05) between the 2 groups, including 11 genera with relative abundance > 1% (*Vibrio*, *Lachnoclostridium*, *Prey Prevotella*, *Roseburia*, *Bacteroides*, *Lachnospira*, *Pseudomonas*, *Lactobacillus*, *Leptotrichia*, *Parabacteroides*, and *Clostridium*). The relative abundances of *Prevotella* and Parabacteroides, belonging to *Bacteroidetes* phyla, were significantly higher in the control group than those in the MA group. However, the other bacterial genera, most of which belong to *Proteobacteria* and *Firmicutes* phyla, showed significantly higher relative abundances in the MA group (*P* < 0.05, Table 3).

**Figure 8:**
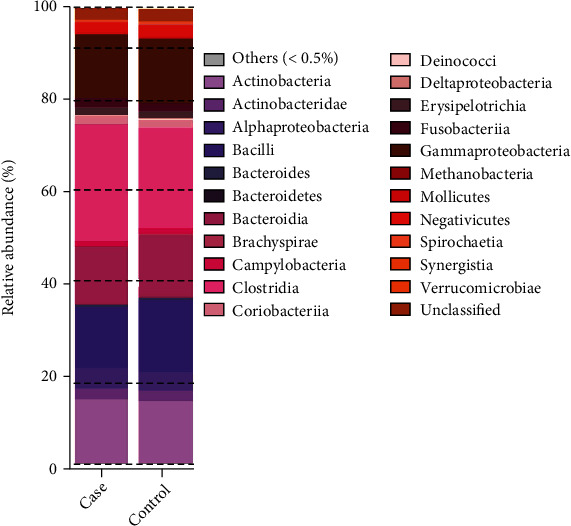
A column chart shows the relative abundances of species in the 2 groups at the genus level. The *P* values of the relative abundance of the intestinal microbiota of the 2 groups are the values after FDR correction.

A detailed classification of the microbial flora was performed at the species level for samples in the 2 groups. More than 1000 species of bacteria were detected in the case group and the control group, among which 457 species showed significant differences (*P* < 0.05, Figure 8).

### 3.4. Potential Functional Pathways Were Predicted through the KEGG Database

**Figure 9:**
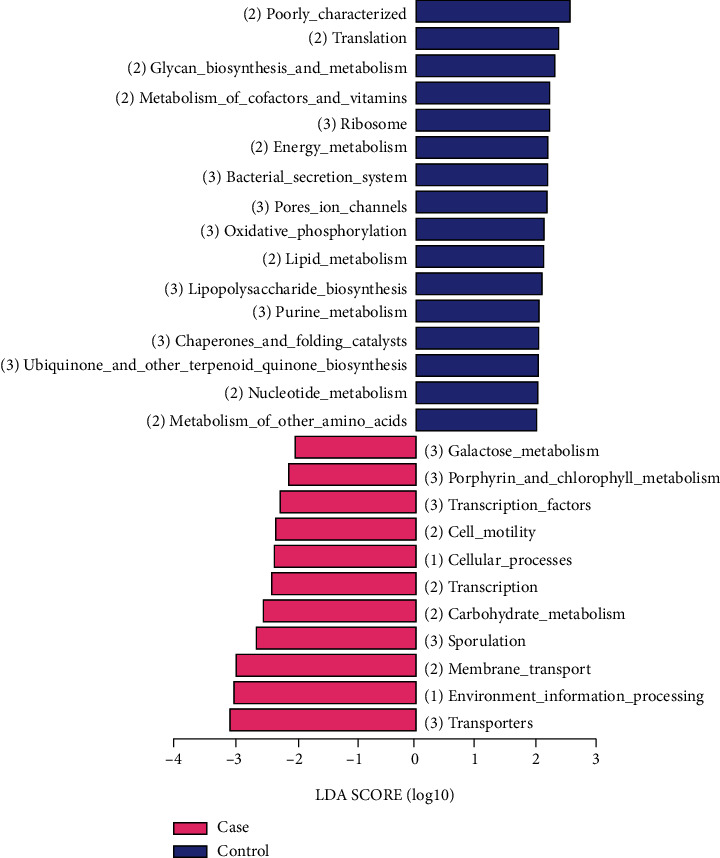
Screening of the differential functions using the KEGG pathway. The abundant signal pathways in the case group are represented by pink bars, and the abundant signal pathways in the control group are represented by blue bars.

The KEGG pathway enrichment analysis revealed that genes expressed by the differentially abundant microbial species were significantly enriched in 27 signaling pathways and metabolic pathways (*P* < 0.05). Most of the potential pathways for the control group were related to energy and nutrient metabolisms (glycan, vitamins, lipid, amino acids, and biosynthesis) and growth and development metabolism (translation, oxidation, catalysts, and nucleotide). Differentially expressed genes involved in pathways related to processing, lipid metabolism, and amino acid metabolism were more abundant in the control group than in the case group (Figure 9).

### 3.5. A Comparison of the Intestinal Mycoplasma

The positive detection rates of *Mycoplasma* and *M. hominis* (MH) were 96% (48 cases) and 38% (19 cases), respectively, in the case group and 98% (49 cases) and 66% (33 cases), respectively, in the control group. There was no statistical difference in the positive detection rate of *Mycoplasma* between the 2 groups (*χ*^2^ = 0.344, *P* = 0.5). However, the positive detection rate of MH was statistically higher in the control group than in the case group (66% vs. 38%; *χ*^2^ = 7.853, *P* = 0.004; Table 4). The average relative abundance of *Mycoplasma* and MH in the control group was significantly higher than that observed in the case group (*P* < 0.05, Table 5).

## 4. Discussion

Abortion is the common complicating disease during pregnancy, affecting about 15% of clinical pregnancies [29]. Recently, many clinical studies indicated that vaginal bacterial composition in the first-trimester miscarriage was associated with reduced abundance of *Lactobacillus* spp., which will lead to the activation of inflammatory pathway and premature delivery [24, 30, 31]. Liu et al. reported that the vaginal bacterial species richness and diversity of women with MAs were higher than those in normal pregnant women in Shanghai, China [32]. The causes of preterm birth and abortion may not be limited to the microbiota in the reproductive tract or local immune response, but rather integrated with maternal systemic immunity.

The gut microbiota is a key factor in the formation and regulation of immune response [33–35]. To further understand the possible relationship between host gut microbiota of women with missed abortions and normal pregnancy, we conducted a clinical study in Hunan, China, and put forward preliminary insights. In our study, the *α*-diversity of gut microbiota in the control group was significantly higher than that of the MAs (*P* < 0.05). The result was the same as that of the reduction of bacterial diversity in the fecal microbiota of miscarriage patients [36]. The decrease in *α*-diversity was still found strongly associated with the development of spontaneous preterm birth [37]. In conclusion, the results of gut microbiota showed that the *α*- and *β*-diversities of women with missed abortions and miscarriage were lower than those of normal pregnant women. Besides, not only the NMDS but also the PCoA predicted the gut microbiota to be significantly different between the case group and the control group (*P* < 0.05), which suggested that the composition of the gut microbiota might have been disturbed, shifted, and rebalanced between the genesis or progression of MAs. This implication is highly consistent with our initial hypothesis, and it was further reinforced by the gut microbiota composition analyses, mainly at the phylum level.

Gut microbiota can digest dietary polysaccharides that cannot be digested by the host into monosaccharides and short-chain fatty acids for intestinal absorption and conversion into lipids in the liver. The greatest change in the gut microbiota occurs in the increase of some key bacteria, which is similar to the high level of *Firmicutes*, butyrate producers, in obese patients [38]. The increase of *Bacteroides* was significantly associated with weight loss but not with total calorie intake. In the first trimester, the gut microbiota pattern is similar to that of healthy nonpregnant women in many aspects, indicating that *Firmicutes* (mainly *Clostridiales*) is dominant over *Bacteroidetes* in our study [39]. In our study, significant differences were found in 8 phyla between the 2 groups (*P* < 0.05). In the capacity for energy harvest, *Firmicutes* were significant enriched in the case group in our study. However, both *Firmicutes* and *Bacteroidetes* were enriched in the miscarriage group compared to the control group in Liu et al.'s study [36]. A previous study also reported that the increased ratio of the *Firmicutes* to *Bacteroidetes* is related to chronic inflammation [21]. The analysis of the *Firmicutes*/*Bacteroidetes* ratio indicated that the change of pathophysiology of obesity occurred in patients with missed abortions, which were similar to those in the miscarriage study. On the other hand, the *Firmicutes*/*Bacteroides* ratio (case : control) in our study (3.29 : 2.92) was higher than that in the miscarriage study (0.65 : 0.80) [36]. The results showed that the nutritional supply of pregnant women was significantly different among regions. Compared with the control group, the IL-2, IL-17A, IL-17, TNF-*α*, and IFN-*γ* levels of serum were increased significantly in the miscarriage group [36]. Although no serological test was performed in our study, it can be inferred from the analysis and comparison above that missed abortion will be highly affected by an immune response and inflammatory reactions.

To support the fetus's growth, the mother's communities of gut microbiota change toward energy production and storage from the first to third trimesters of pregnancy. At the phylum level, *Firmicutes* have been associated with increased energy storage, whereas *Proteobacteria* and *Actinobacteria* protect mother and fetus from external infections. *Actinomycetes* have unparalleled metabolic diversity, and their secondary metabolites can be used as clinical antibiotics, anticancer compounds, immunosuppressants, and so on [33]. During the chronic inflammation (aging process) of female mice, the abundance of *Actinobacteria* and *Firmicutes* increased, while the *Proteobacteria* decreased. In our study, the abundance of *Actinobacteria*, *Firmicutes,* and *Proteobacteria* increased in patients with missed abortions. Only *Actinobacteria* was found significantly higher in MAs than in control groups, but the finding cannot be observed in miscarriage and premature delivery groups [36, 37]. This is the first report of the high abundance of *Actinobacteria* found in the feces of pregnant women with missed abortions. The reasonable explanation is that mothers need more mechanisms to protect themselves from infection caused by embryonic stagnation or stillbirth. The increase of *Actinobacteria* in the gut of the mother illustrates one of the possible processes to protect the mother far from infection.

*Proteobacteria* may be the possible microbial signature of metabolic disorders and inflammatory bowel disease. The higher intake of monounsaturated fat, cholesterol, and fat-soluble vitamins was associated with increased *Proteobacteria*. Most clinical studies have focused on the increase of *Proteobacteria* in the gut microbiota of patients with inflammatory bowel diseases. Our study found that the relative abundance of *Proteobacteria* in the MA group was 3% higher than that in the control group. Liu et al. showed *that Proteobacteria* was enriched in the normal group, not the miscarriage group [36]. The difference in the 2 studies can be explained that the increase of *Proteobacteria* might be related to embryonic stagnation or different inflammatory genesis to protect the mother. This might be the first attempt to explore the relationship between gut *Proteobacteria* and the MAs. The abundance of *Proteobacteria* was related to the disease progression of the genital and respiratory systems, but it may also be related to the gut system for MAs. From the study, the abundance of *Proteobacteria* and *Actinobacteria* was found in the MA group, which can be inferred as a possible mechanism to protect mothers from internal infection caused by dead fetuses. In conclusion, this is the first time that the simultaneous increase of *Proteobacteria* and *Actinobacteria* has been found in pregnant women, which can speculate that the increase of specific intestinal flora may be related to the avoidance of infection, immunity, and inflammation.

Two significant abundances of bacterial genera, *Prevotella* and *Parabacteroides*, belonged to *Bacteroidetes* phyla. The other 9 significant abundances of bacterial genera in the case group were classified in *Firmicutes* (5 phyla), *Proteobacteria* (2 phyla), and *Bacteroidetes* (1 phylum). Therefore, there is more energy metabolizing relative gut microbiota found in the gut of normal pregnant women, while more energy accumulation and defense-related bacteria were found in women with missed abortions. The conclusion from gut microbiota is similar to the results of the KEGG pathway (Table 3). Gut microbial dysbiosis is a risk factor in the development of inflammation [40, 41]. The potential mechanism of our findings might be addressed as the disturbed gut microbiota accumulating secretions such as the hepatic fibroblast growth factor 21 (FGF21) through the gut-liver axis or bile metabolism leads to the impaired lipid metabolism [34, 42], triggers the intestine inflammation via the NF-KB pathway activation [35, 43], further damages the intestine barrier [43], destroys the energy balance, and finally alters the systematic homeostasis [44]. To the best of our knowledge, this is the first study to investigate the association of gut microbiota and lipid/inflammation metabolism in MA patients. This may contribute to the interplay between the host and gut microorganisms in terms of metabolism modulation.

The gut microbiota of the patients in this study was further investigated at the species level. Out of 1000 bacteria species examined, 457 were found to be significantly different between the two groups (*P* < 0.05). However, the relative abundance of bacterial species was less than 0.1% of the total species in the gut. We infer that changes in species of gut microbiota do not affect outcomes and prognosis in our study.

The relative abundance of vaginal *Lactobacillus* was significantly increased in the control group compared to the risk of early pregnancy miscarriage, which was supported by the 2007 study by Nelson et al. [32, 45]. It was also reported that the decrease in the abundance of vaginal *Lactobacillus* spp. drives the activation of inflammatory pathways, thereby reducing endometrial receptivity and implantation. Lower *Lactobacillus spp.* might increase the richness and diversity of potential pathogens, such as *Prevotella* and *Mycoplasmas* in the vaginal bacterial ecosystem [46]. However, our data of gut microbiota suggested that the relative abundance of *Prevotella*, *Mycoplasma*, and MH was significantly higher in the control group compared to the MA group. This may be the differences in disease correlation caused by the location of microbiota communities or differences in the metabolic mechanisms of different anatomical systems [47].

There were certain limitations in this investigation exploring the potential correlation between the gut microbiota and the MAs. First, the relatively small sample size in this study may not represent the general population. Second, the gut microbial composition depends on perturbations, such as dietary habits, antibiotics, age, sanitation, hygiene, geography, climate, environment exposure, and health conditions [48–55]. We did not obtain a complete questionnaire on participants' dietary habits, so we were unable to determine whether the diet was a factor in the gut bacterial imbalance. In addition, we cannot rule out other etiologies of miscarriage patients, such as genetic variation. Then, the related serum biomarkers of the MAs were not collected, tested, and compared. Therefore, we found the possible metabolic pathways but did not further evidence for the relevant metabolites of potential pathways. Therefore, a more extensive and multicentered study with detailed questionnaires, metabolites, and genetic variation would benefit future investigations. However, this is the first clinical study to provide significant findings and new insights into the imbalance of gut microbiota associated with missed abortion.

In conclusion, we demonstrated an association between gut bacterial dysbiosis and MAs. An enormous diversity of gut flora has a symbiotic relationship with the health of an individual. It is the first report that the protection bacteria, *Proteobacteria* and *Actinobacteria*, have significant abundance in MAs but not in patients with miscarriage or premature delivery. It could be the internal mechanism to protect the mother far from infections. This study provided insights into the potential change of gut microbiota of MAs and the potential underlying mechanisms through certain impaired lipid metabolism and aroused inflammation pathways. Understanding the interaction between gut microbiota and MAs will contribute to the conception and realization of novel diagnostic, therapeutic, and preventive strategies for MAs. Moreover, the structural manipulation of gut microbiota communities may be a promising target for regulating metabolic balance in patients with missed abortions.

## Figures and Tables

**Table 1:** Characteristics of the case group and the control group.

General information	Case group	Control group	*t*/*z*/*χ*2	*P* value
Age (years)	31.7 ± 4.6	30.9 ± 4.1	-0.93	0.3568
Menstrual flow			0.7092	0.3997
No change	46 (92%)	48 (96%)		
Reduced	4 (8%)	2 (4%)		
Gravidity (times)			3.5088	0.061
0	8 (16%)	16 (32%)		
1	10 (20%)	19 (38%)		
≥2	32 (64%)	15 (30%)		
Parity (times)			0.3613	0.5478
0	25 (50%)	22 (44%)		
≥1	25 (50%)	28 (56%)		
Abortion (times)			18.5372	<0.0001
0	9 (18%)	30 (60%)		
1	24 (48%)	13 (26%)		
≥2	17 (34%)	7 (14%)		
Intrauterine operation			16.9779	<0.0001
No	9 (18%)	28 (56%)		
Yes	41 (82%)	22 (44%)		
Cesarean section			0.0486	0.8256
No	36 (72%)	35 (70%)		
Yes	14 (28%)	15 (30%)		

*P* value > 0.05 represents no significant difference; *P* value < 0.05 represents significant difference. Intrauterine operation: intrauterine surgeries such as abortion and hysteroscopic procedures. Cesarean section: a surgical procedure used to deliver a baby through incisions in the abdomen and uterus.

**Table 2:** The statistics of the *α*-diversity analyses.

*α*-Diversity	Average (case)	SD value (case)	Average (control)	SD value (control)	*P* value
Sobs	663.48000	231.67115	837.96000	232.63302	0.00077
Chao	663.48000	231.67115	837.96000	232.63302	0.00077
ACE	663.48000	231.67115	837.96000	232.63302	0.00077
Shannon	5.49967	0.70135	5.89128	0.90201	0.00185
Simpson	0.01692	0.03893	0.01296	0.01042	0.00576

Sobs, Chao, ACE, Shannon, and Simpson are types of *α*-diversity indexes. *P* value > 0.05 represents no significant difference; *P* value < 0.05 represents significant difference. Sobs: number of observed species; ACE: abundance-based coverage estimator; SD: standard deviation.

**Table 3:** A comparison of the differential bacterial genera between the 2 groups.

Differential bacteria	Case	Control	*P* value
*Vibrio*	1.1886	0.9978	0.0187
*Clostridium*	1.3399	0.9580	0.0010
*Prevotella*	0.7007	1.4912	0.0112
*Roseburia*	1.5566	1.1781	0.0044
*Bacteroides*	4.4468	3.9660	0.0367
*Laospirillum*	1.2569	0.8197	0.0011
*Pseudomonas*	1.2058	1.0328	0.0463
*Lachnospira*	1.2746	2.0662	0.0046
*Ciliates*	1.5743	1.1802	0.0406
*Parabacteroides*	1.1873	1.4232	0.0399
*Bacillus*	1.1831	0.3344	0.0005

*P* value > 0.05 represents no significant difference; *P* value < 0.05 represents significant difference.

**Table 4:** A comparison of the positive detection rate of *Mycoplasma* between the 2 groups.

*Mycoplasma*	Case group [*n* (%)]	Control group [*n* (%)]	*χ* ^2^	*P* value
*Mycoplasma*	48 (96%)	49 (98%)	0.3440	0.5000
*Mycoplasma hominis*	19 (38%)	33 (66%)	7.8530	0.0040

*P* value > 0.05 represents no significant difference; *P* value < 0.05 represents significant difference.

**Table 5:** A comparison of the average relative abundance of *Mycoplasma* between the 2 groups.

*Mycoplasma*	Case	Control	*P* value
	(AVG ± SD)	(AVG ± SD)	
*Mycoplasma*	0.2306 ± 0.1714	0.3009 ± 0.1592	0.0360
*Mycoplasma hominis*	0.0202 ± 0.2873	0.0392 ± 0.3628	0.0040

Note: *P* value > 0.05 represents no significant difference; *P* value < 0.05 represents significant difference. AVG: average; SD: standard deviation.

## Data Availability

The patients' data used to support the findings of this study are documented in our department.
